# Postural Control in Bipolar Disorder: Increased Sway Area and Decreased Dynamical Complexity

**DOI:** 10.1371/journal.pone.0019824

**Published:** 2011-05-18

**Authors:** Amanda R. Bolbecker, S. Lee Hong, Jerillyn S. Kent, Mallory J. Klaunig, Brian F. O'Donnell, William P. Hetrick

**Affiliations:** 1 Department of Psychological and Brain Sciences, Indiana University, Bloomington, Indiana, United States of America; 2 Larue D. Carter Memorial Hospital, Indianapolis, Indiana, United States of America; 3 Department of Kinesiology, Indiana University, Bloomington, Indiana, United States of America; 4 Department of Psychiatry, Indiana University School of Medicine, Indianapolis, Indiana, United States of America; The University of Hong Kong, Hong Kong

## Abstract

Structural, neurochemical, and functional abnormalities have been identified in the brains of individuals with bipolar disorder, including in key brain structures implicated in postural control, i.e. the cerebellum, brainstem, and basal ganglia. Given these findings, we tested the hypothesis that postural control deficits are present in individuals with bipolar disorder. Sixteen participants with bipolar disorder (BD) and 16 age-matched non-psychiatric healthy controls were asked to stand as still as possible on a force platform for 2 minutes under 4 conditions: (1) eyes open-open base; (2) eyes closed-open base; (3) eyes open-closed base; and (4) eyes closed-closed base. Postural sway data were submitted to conventional quantitative analyses of the magnitude of sway area using the center of pressure measurement. In addition, data were submitted to detrended fluctuation analysis, a nonlinear dynamical systems analytic technique that measures complexity of a time-series, on both the anterior-posterior and medio-lateral directions. The bipolar disorder group had increased sway area, indicative of reduced postural control. Decreased complexity in the medio-lateral direction was also observed for the bipolar disorder group, suggesting both a reduction in dynamic range available to them for postural control, and that their postural corrections were primarily dominated by longer time-scales. On both of these measures, significant interactions between diagnostic group and visual condition were also observed, suggesting that the BD participants were impaired in their ability to make corrections to their sway pattern when no visual information was available. Greater sway magnitude and reduced complexity suggest that individuals with bipolar disorder have deficits in sensorimotor integration and a reduced range of timescales available on which to make postural corrections.

## Introduction

Although the nature and origins of bipolar disorder (BD) are still relatively poorly understood, abnormalities in diverse brain regions have been identified. Behavioral, structural, and diffusion tensor imaging studies provide convergent evidence of anterior limbic network abnormalities in BD, and the pattern of emotional and cognitive deficits observed in BD is consistent with abnormalities in a cerebello-striatal-prefrontal circuit [1], [2].

An emerging literature suggests motor abnormalities accompany mood and psychotic symptoms of BD, although the relationship between motor and mood disorders has rarely been studied explicitly [3]. Some motor symptoms appear to be state-related, i.e., linked to either manic, depressed, or mixed mood states, while other symptoms, such as tardive dyskinesia and myoclonus emerge from the use of neuroleptic medications [4]. Accumulating evidence indicates that subtle motor anomalies may exist independent of acute mood state in BD and motor dysfunction could, therefore, be a core feature of the disorder. Such neurological soft signs have been observed to be significantly increased in euthymic BD patients in comparison to controls [5], [6], [7].

Importantly, the brain areas that participate in mood regulation and have been found to be abnormal in BD also play critical roles in motor function. For example, the cerebellum is a key structure in motor control and plays an integral role in the production of smooth, coordinated movement and in maintaining postural control through appropriately timed activation of agonist and antagonist muscles. More recently, empirical and theoretical evidence have indicated that the cerebellum plays a significant role in psychological functions as well, including modulation of perceptual, cognitive, and affective functions [8], [9], [10], [11], [12], which is believed to occur via its modulation of the anterior limbic network [1], [13], [14], [15]. Structural imaging studies indicate cerebellar abnormalities, in particular, cerebellar atrophy in BD [13], [16], [17], [18], [19], [20], [21], [22]. Neurochemical alterations have also been reported [23], [24], [25], [26]. Moreover, behavioral evidence also points to disturbances in cerebellar function in people with BD, who exhibit deficits in eyeblink conditioning, a sensitive assay of cerebellar function [27].

The basal ganglia also play a crucial role in motor behavior and show abnormalities in BD. This brain circuit is crucial for the initiation of movement and plays an important role in multisensory integration, especially proprioceptive-motor integration [28]. This latter function is particularly critical for postural control. Neuroimaging evidence suggests alterations in the basal ganglia of individuals with BD [29], [30], [31]. Behavioral evidence also supports basal ganglia dysfunction in BD. For example, BD patients were significantly impaired in a study of two electromechanical measures of motor function, force steadiness, and velocity scaling, which are sensitive to basal ganglia abnormalties [3].

Finally, the brainstem is also critically involved in motor function, and is particularly involved in the coordination of vestibular and visual input with afferent proprioceptive information [32]. Several small studies have reported abnormalities in the brainstem nuclei of BD patients, particularly in the locus coeruleus [33], [34].

Postural sway is a sensitive test of the integrity of motor control that is likely to be affected by abnormal or aberrant functioning of the cerebellum, basal ganglia, and brainstem. Given evidence of abnormalities in the aforementioned brain circuits in bipolar disorder, the present study tested the hypothesis that BD patients exhibit increased postural sway, indicative of poorer postural regulation, relative to a healthy control group. The second goal of this research was to test the hypothesis that the dynamic properties of movement as it evolves over time are also abnormal in BD. To examine the processes generating the sway pattern, dynamic analyses were applied [35]. Complexity theory in health [36] predicts that disease states manifest themselves through a loss of complexity, that is, a shift from irregularity to greater regularity. This shift would be manifested in a sway pattern that evolves primarily on slower time-scales due to the loss of high frequency components in the system, which allow for faster and smaller-scale postural adjustments. Indeed, this increase in regularity of movement has been observed in several clinical populations [37]. In contrast, the sway patterns of healthy people would be predicted to possess a broader range of time-scales, which allows for greater behavioral adaptability. Loss of complexity is hypothesized to be a reflection of a decline in the number of components or connections between these components, for example, the availability and integration of different sources of sensory information [38], [39]. In the current context, such a change in sway pattern could be indicative of a deficit in in multisensory integration mediated by cerebellar, basal ganglia, and brainstem circuits.

In order to examine the amount and dynamic pattern of postural sway in participants with BD, four different postural conditions that alter the availability of proprioceptive (closed vs. open base stance) and visual (eyes open vs. eyes closed) information were employed. Proprioceptive, vestibular, and visual inputs affect different time-scales contributing to the correction of postural stability and removal of any one of these components cause increases in sway area [40]. For example, visual cues stabilize posture on longer time-scales [41], [42], whereas proprioceptive cues are responsible for short timescale corrections [43]. Therefore, if deficits in postural control exist in BD, manipulations of sensory input may be revealing with respect to specific domains in which sensory integration is affected. It was hypothesized that sway area would be significantly larger in BD in comparison to a non-psychiatric healthy control group. Moreover, it was expected that BD participants would be more affected by a change in stance and the loss of visual input, manifested as increased sway area, reflecting decreased integration of sensorimotor information.

Finally, although sway area generally can be expected to increase with the removal of sensorimotor input, the alterations in the complexity of sway dynamics caused by manipulations of either proprioceptive or visual input in non-clinical populations are not identical. For example, the proprioceptive feedback loop works along much short time-scales [10], whereas the visual system contributes to low frequency, longer time-scale postural control [41], [42]. Detrended fluctuation analysis (DFA) [44] was employed to quantify the architecture of spatiotemporal patterns resident in postural sway as they unfold over time, and to examine how manipulations of visual and proprioceptive input altered this architecture. Essentially, DFA quantifies the relationship between variability and the timescale on which it is measured. The primary DFA output is the α-value, where higher values generally indicate decreased complexity and lower values reflect increased complexity. Reduction of proprioceptive input could be expected to reduce the overall complexity of postural regulation and increase DFA α-values due to reduced high frequency, short time-scale components in the postural sway pattern. In contrast, removal of visual input should increase the complexity of postural corrections, resulting in lower DFA α-values. Therefore, the examination of the dynamical properties of postural sway using DFA may provide information regarding whether specific aspects of sensorimotor integration are affected in bipolar disorder. We predict that, consistent with the loss of complexity hypothesis, DFA of postural sway in BD patients will reveal decreased complexity overall and therefore be less affected by alterations in the amount of sensorimotor information available, indicating that postural control is predominated by long time-scale components and reflecting less behavioral flexibility in the motor control domain.

## Methods

### Ethics statement

The study procedures were approved by the Indiana University-Purdue University Indianapolis Institutional Review Board and the study was conducted in accordance with the Declaration of Helsinki (Edinburgh amendments). Written informed consent was obtained from all participants.

### Subjects

Participants included in the analyses were 16 individuals (7 women) with DSM-IV bipolar disorder (BD) and 16 age-matched non-psychiatric healthy controls (9 women). A boxplot method of outlier identification (SPSS statistical package) was used to classify extreme data values separately for each analysis. Extreme outliers were defined as data values>6 quartiles from the upper or lower ends of the inter-quartile range. Following age-matching, there were initially 18 participants in each group, but one BP and one control were removed from the analysis due to classification as extreme outliers in at least one COP condition. All demographic and statistical information is reported for the remaining 16 participants in each group. Gender did not differ between groups (X^2^(1) = 0.50, p = ns). Diagnostic status was determined using the Structured Clinical Interview for DSM-IV Axis I Disorders (SCID-I) [45] sections for mood disorders, psychotic disorders, and substance abuse disorders, and chart review. BD patients were enrolled in a longitudinal study in which their mood was assessed using the SCID-I as well as clinical symptom ratings. The Young Mania Rating Scale (YMRS) [46] was used to assess symptoms of mania and Montgomery-Åsberg Depression Rating Scales (MADRS) [47] was used to evaluate depressive symptoms. All BD participants were in a euthymic state when they participated in the postural sway experiment. Healthy controls were recruited through newspaper advertisements and fliers, and did not meet DSM-IV criteria for any Axis I or Axis II disorder. Any participant who met criteria for substance dependency within three months prior to testing was excluded from the study. Diagnostic interviews and clinical ratings were performed by trained research personnel. Kappa inter-rater reliability in this laboratory setting has been 0.95 for mood disorders vs. schizophrenia, or other diagnoses.

The mean age of BD participants (38.6 yrs, SD = 10.5) did not differ from controls (38.4 yrs, SD = 10.5), t(30) = −0.07, p = ns. Body mass index (BMI) of BD participants (M = 27.9, SD = 5.2) and controls (M = 27.6, SD = 5.8) also did not statistically differ, t(30) = −0.16, p = ns. Inclusion criteria were completion of grade school level education, normal or corrected to normal hearing and vision, no history of cardiovascular or neurological disease, body mass index of less than 40, and no history of head injury that resulted in loss of consciousness. All BD participants were euthymic, with mean YMRS scores of 4.6 (SD = 5.1) and MADRS scores of 4.2 (SD = 4.7). Finally, BD participants had been assessed within the previous 2 weeks using the Abnormal Involuntary Movement Scale (AIMS) [48]. No participants had positive AIMS scores.

Four individuals with bipolar disorder were un-medicated at the time of testing. The remaining 12 were on various combinations of psychotropic medications, which are listed for each individual in Table 1.

**Table 1:**
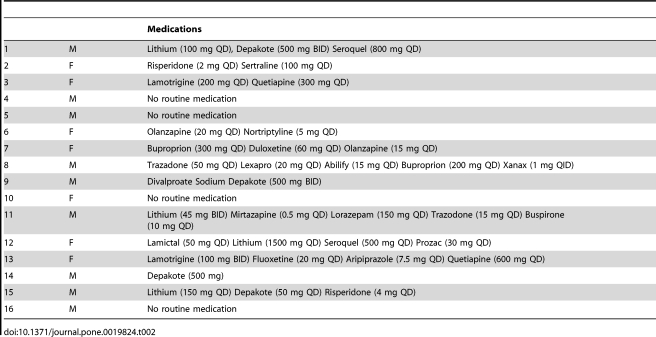
Detailed List of scheduled psychotropic medications for bipolar disorder participants.

### Task and Procedures

Each participant was required to stand as still as possible while barefoot on an AMTI Accusway (Watertown, MA) force platform under the following task conditions: (1) eyes open-open base; (2) eyes closed-open base; (3) eyes open-closed base; and (4) eyes closed-closed base. During the open base conditions, feet were placed shoulder width apart; participants stood with their feet together (approximately 1 inch apart) during the closed base conditions. Each trial lasted 2 minutes.

### Data Analysis

**Figure 1:**
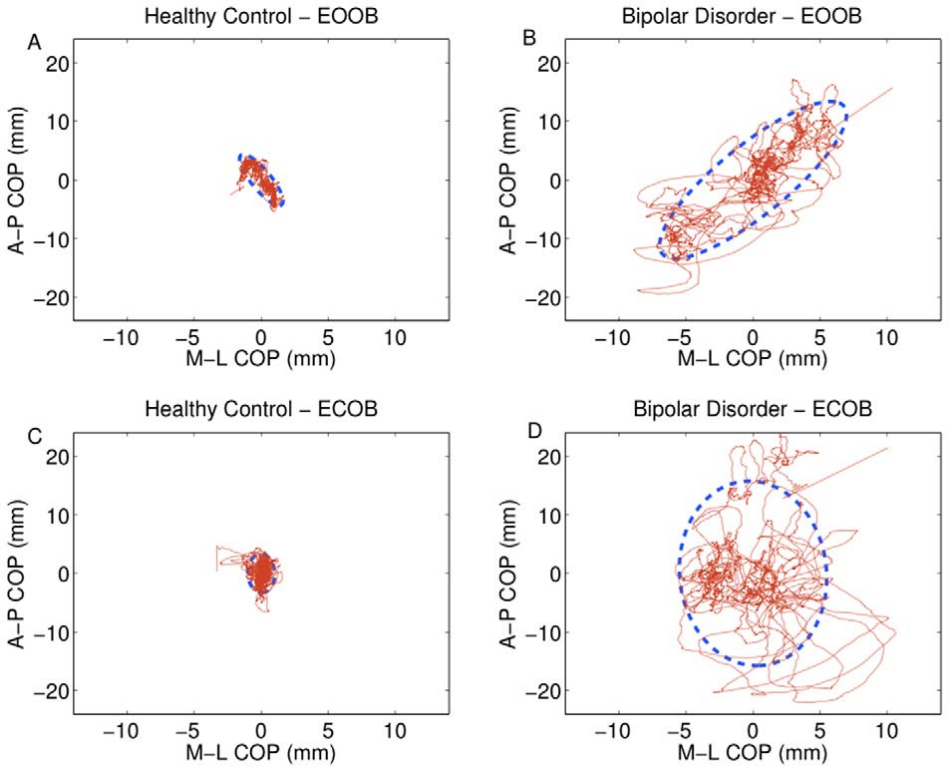
Exemplar plots for COP in a healthy control and a bipolar disorder participant. Sway path is in red, with sway area represented in blue. Eyes open-open base sway areas are shown for the control (A) and bipolar disorder (B) participant. Corresponding data for the eyes closed-open base condition are shown in the lower panels for the same control (C) and bipolar disorder (D) participants.

The center of pressure (COP) motion along the anterior-posterior and medio-lateral axes of motion were obtained from the force platform, sampled at a rate of 50 Hz and filtered with a 9th order Butterworth low-pass filter with a 25 Hz cutoff frequency to isolate the low-frequency postural sway process. Sway area was measured during each trial to provide the amount of sway for each participant during each condition. Postural sway signals have time-varying statistical properties [49], which is reflected in the fact that taking the average at different time points during the task results in a “wandering mean”. These variations in mean and standard deviation over time are known as nonstationarity. To minimize the effects of nonstationarity in the postural sway time series, a 95% confidence ellipse was obtained around the COP motion along both the anterior-posterior and medio-lateral axes using the method presented in Oliveira et al. [50], as this method is much more robust to the effects of outliers. Exemplar data from a BD and a control participant are depicted graphically in Figure 1 with corresponding confidence ellipses for the eyes open and eyes closed conditions in the open stance condition.

**Figure 2:**
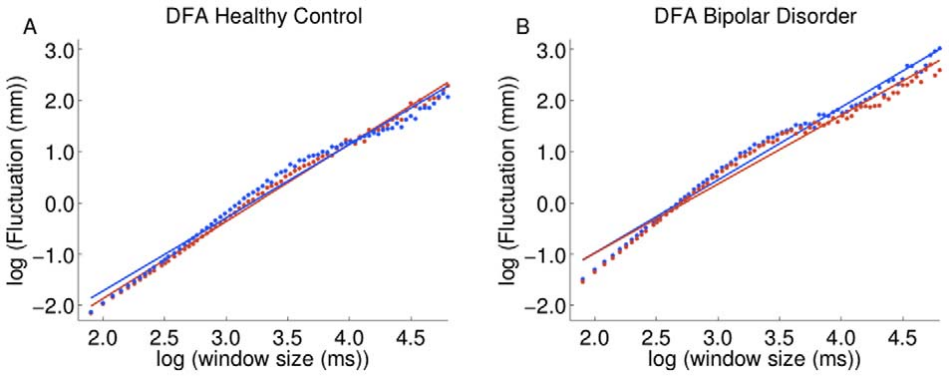
Detrended fluctuation analysis for derived from the exemplar COP data shown in **Figure 1** for the eyes open-open base condition (blue) and the eyes closed-open base condition (red). Data for the control and bipolar disorder participant can be found in Panel A and B, respectively. Each individual symbol in each panel plots the fluctuation magnitude against the particular timescale on which it is measured. The slope of the fitted line for each condition produces the α-value, which is the primary dependent variable for DFA.

In order to assess the more complex dynamics of postural sway, detrended fluctuation analysis (DFA) was performed on the COP data. DFA was specifically designed to be robust against nonstationarity within a time-series [44] and therefore represents a superior approach to traditional descriptors of variability such as the standard deviation. The DFA analysis indexes the relative distribution of variance within the data across a range of different time-scales. This produces a profile of the time series in terms of the rate of growth in fluctuation of variance as a function of increasing time-scale. The rate of growth in fluctuation magnitude across time-scales is indexed by the slope of this function (plotted on a log-log scale), known as the α-value, which is an index of long-range autocorrelations in time-series. The plotted DFA for a single subject is shown in Figure 2. An α-value of 1 is present in 1/*f* noise and characterizes fractals and healthy physiological systems, indicating the maximum degree of self-similarity in a signal [51]. This is a unique pattern of complexity, as the magnitude of the fluctuations grows in direct proportion to the time-scale on which the fluctuations are measured. A time series characterized by fluctuations across fewer time scales would yield a steeper slope, i.e. a larger α-value, indicating a less complex system. A flatter slope, i.e., lower α-value indicates that fluctuations are spread more evenly across a range of time scales in the time series, reflective of greater complexity. It is important to note, however, that values of DFA of 0.5 indicate a completely random, or white noise process, while values <0.5 represent an anti-persistent time-series, where the behavior of the system at future time points is antagonistic to that of its past and present. DFA was calculated for both side-to-side, or medio-lateral (ML), and front-to-back, or anterior-posterior (AP), directions. Due to a main effect of direction (ML versus AP) and an interaction between direction and diagnostic group (F(1,34) = 6.44, p<.05) in the detrended fluctuation analysis (F(1,34) = 32.35, p<0.001), separate statistical analyses were conducted for the α-values calculated for medio-lateral sway (DFA-ML) and antero-posterior (DFA-AP) sway.

The three dependent variables (sway area, DFA-ML, and DFA-AP) were evaluated using a 2 (Vision: eyes open vs. eyes closed)×2 (Base: open base vs. closed base)×2 (Group: BD vs. control) Repeated Measures ANOVA. Time, Vision, and Base were within-subjects factors while Group served as the between-subjects factor. To evaluate possible medication effects on postural sway performance, participants with bipolar disorder were collapsed into a single group with medication status as the independent variable. Participants were divided into three groups: those on antipsychotic medication (typical or atypical) were assigned to the “antipsychotic” group (n = 9), those who were on other psychotropic drugs but were not taking antipsychotic medication were assigned to the “other psychotropic” category (n = 3), and those who were not currently taking medication were included in the “unmedicated” group (n = 4). Repeated measures ANOVAs were then conducted for all primary dependent variables. In addition, bipolar disorder participants were coded as “on” or “off” for the following medication categories: atypical antipsychotic drug use (ON = 9), SSRIs (ON = 4) and a test of medicated (any psychotropic medication including antipsychotics) versus unmedicated participants (ON = 12). Separate ANOVAs were conducted for each category and for each dependent variable. Finally, chlorpromazine equivalent dosages were calculated using the method described by Woods [51].

Results of the major dependent variables are reported with their corresponding effect sizes in the form of partial eta^2^ (ηP^2^). An estimate of effect size was provided by Cohen [52]: small effect sizes are less than 0.06; moderate effect sizes range from 0.06 to 0.14; large effect sizes are greater than .14. The α-level was set at p<0.05. Post-hoc univariate tests were conducted for significant (p<0.05) interactions.

## Results

Exemplar plots of COP data from a BD and control participant in the eyes-open and eyes-closed conditions in an open stance are shown in Figure 1, with corresponding plots of their DFA graphically depicted in Figure 2. These particular participants were chosen because their COP data were closest to the means within their groups. Group means and standard deviations for each dependent variable can be found in Table 2.

**Table 2:** Means and standard deviations for COP, DFA-ML and DFA-AP for bipolar disorder and healthy control groups.

		Medications
1	M	Lithium (100 mg QD), Depakote (500 mg BID) Seroquel (800 mg QD)
2	F	Risperidone (2 mg QD) Sertraline (100 mg QD)
3	F	Lamotrigine (200 mg QD) Quetiapine (300 mg QD)
4	M	No routine medication
5	M	No routine medication
6	F	Olanzapine (20 mg QD) Nortriptyline (5 mg QD)
7	F	Buproprion (300 mg QD) Duloxetine (60 mg QD) Olanzapine (15 mg QD)
8	M	Trazadone (50 mg QD) Lexapro (20 mg QD) Abilify (15 mg QD) Buproprion (200 mg QD) Xanax (1 mg QID)
9	M	Divalproate Sodium Depakote (500 mg BID)
10	F	No routine medication
11	M	Lithium (45 mg BID) Mirtazapine (0.5 mg QD) Lorazepam (150 mg QD) Trazodone (15 mg QD) Buspirone (10 mg QD)
12	F	Lamictal (50 mg QD) Lithium (1500 mg QD) Seroquel (500 mg QD) Prozac (30 mg QD)
13	F	Lamotrigine (100 mg BID) Fluoxetine (20 mg QD) Aripiprazole (7.5 mg QD) Quetiapine (600 mg QD)
14	M	Depakote (500 mg)
15	M	Lithium (150 mg QD) Depakote (50 mg QD) Risperidone (4 mg QD)
16	M	No routine medication

	EOOB	EOCB	ECOB	ECCB
**COP Area**Healthy ControlsBipolar Disorder	11.5 (6.9)44.1 (46.6)	22.2 (11.7)85.9 (85.9)	12.7 (6.4)76.4 (92.4)	40.8 (22.6)146.0 (148.8)
**DFA-ML (α-values)**Healthy ControlsBipolar Disorder	1.22 (0.13)1.27 (0.06)	1.33 (0.10)1.36 (0.09)	1.19 (0.13)1.30 (0.12)	1.28 (0.10)1.35 (0.09)
**DFA-AP (α-values)**Healthy ControlsBipolar Disorder	1.35 (.07)1.36 (.11)	1.40 (.07)1.38 (.08)	1.35 (.07)1.37 (.09)	1.31 (.10)1.31 (.11)

EOOB: eyes open-open base; EOCB: eyes open-closed base; ECOB: eyes closed-open base; ECCB: eyes closed-closed base. COP: Center of Pressure; DFA-ML: Dentrended Fluctuation Analysis-Medio-Lateral direction; DFA-AP: Dentrended Fluctuation Analysis-Anterior Posterior direction.

### Medication analysis

No significant differences for medication status were found for any primary dependent variables, nor were there significant correlations between chlorpromazine equivalent dosages and any postural sway variables.

### Alcohol and Substance Use

Although no participants with current alcohol dependence were included in the study, 5 participants with bipolar disorder had previously met criteria for DSM-IV Alcohol Dependence. When these participants were excluded and all analyses were run including only the remaining 11 who had no history of alcohol dependence, all results involving interactions with and main effects of diagnosis reported below were essentially unaltered. All significant results using the entire sample continued to reach significance (p<0.05).

### Center of Pressure Area

**Figure 3:**
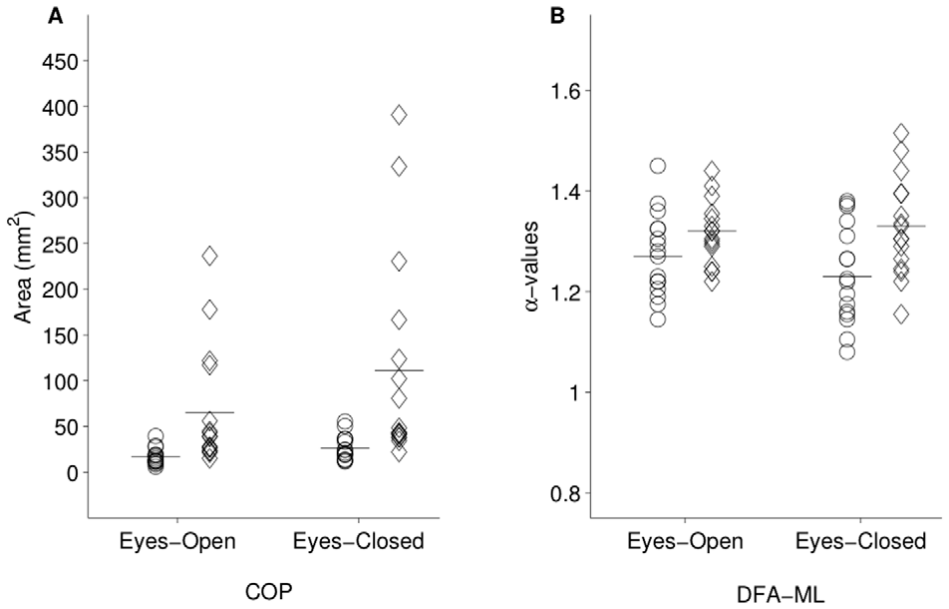
Vision x diagnosis interactions for COP (Panel A) and DFA-ML (Panel B). Each participant's data is represented by a single data point in the eyes-open and eyes-closed condition in each panel. COP increased more dramatically for bipolar disorder participants (diamonds) than for controls (circles). DFA-ML decreased for controls, but was relatively unaffected in bipolar disorder, suggesting that the patient group was less able to compensate for the loss of visual information by using vestibular or proprioceptive information.

The bipolar disorder group had significantly larger sway areas than controls, resulting in a main effect of diagnosis, (F(1,30) = 9.08, p<0.01 (ηP^2^ = 0.23). There was also an interaction between visual condition and diagnostic group, F(1,30) = 5.64, p<0.05 (ηP^2^ = 0.16), due to the BD group showing increased sway compared to the control group in the eyes closed condition. Figure 3A graphically depicts the changes in both groups as a function of visual input. A marginally significant base x diagnosis interaction was observed, F(1,30) = 4.10, p = 0.05 (ηP^2^ = 0.12), due to the BD group showing an increase in sway area in the closed base condition compared to controls. A significant within-subjects visual condition x stance interaction was also apparent (F(1,30) = 7.08, p<0.05 (ηP^2^ = 0.19), in which the eyes closed condition had a larger effect on the closed base condition relative to the open stance condition. There were also within-subjects main effects of visual condition, F(1,30) = 13.50, p = 0.001, ηP^2^ = 0.31, and stance, F(1,30) = 17.60, p<0.001, ηP^2^ = 0.37, where sway areas were smaller during the eyes open and open stance conditions.

### Detrended Fluctuation Analysis: Medio-Lateral

The DFA analysis yielded a significant main effect of diagnosis (F(1,30) = 5.71, p<0.05, η_P_ = 0.16), due to higher α-values overall in the BD group. In addition, there was a vision x diagnostic group interaction (F(1,30) = 5.54, p<0.05, η_P_ = 0.16). A post-hoc analysis of the vision x diagnosis interaction revealed a significant difference between diagnostic groups during the eyes-closed conditions (p<0.05) where controls had lower α-values than the BD participants. DFA results for both groups in the eyes open and eyes closed conditions can be seen in Figure 3B. This difference was not observed when the participants' eyes were open. A significant within-subjects effect of stance was observed, F(1,30) = 22.78, p<0.001, η_P_ = 0.43, where α-values were lower during the open-base conditions in comparison to the closed-base conditions.

### Detrended Fluctuation Analysis: Anterior-Posterior

There were no significant interactions or main effects for diagnostic group (p>0.05). A significant within-subjects vision x stance interaction was observed, (F(1,30) = 34.88 p<0.001, η_P_^2^ = .54) as well as a significant effect of vision (F(1,30) = 19.23, p<0.001, η_P_^2^ = .39). Post-hoc analysis of the vision x stance interaction revealed that α-values were significantly lower (p<0.05) in the eyes open condition when the base was open compared to closed; however, α-values were significantly higher (p<0.01) in the eyes closed condition when the base was open compared to when it was closed. The vision effect showed that α-values were higher when the participants' eyes were open.

## Discussion

The primary findings of the present study were that participants with bipolar disorder manifested increased postural sway in comparison to non-psychiatric controls and were particularly affected by the loss of visual information. Our finding of greater sway across the various stances and vision conditions suggests poorer postural control in bipolar disorder and is consistent with previous findings of motor dysfunction in BD [3], [5], [6], [7].

A key finding was that the loss of visual information in the eyes closed condition resulted in increased sway area in the bipolar disorder group, an effect not observed in controls. This suggests that the BD participants have reduced postural control when visual information is absent. Interestingly, the narrowing of the stance, which reduces the availability of proprioceptive information, did not compound the effects of reduced visual information in BD participants. Our results also show that increasing the difficulty of the postural task does not necessarily magnify differences between controls and participants in the bipolar disorder group insofar as there were no significant post-hoc group differences (p>0.05) in the most challenging stance and vision conditions (i.e., the eyes-closed and closed-base position).

When dynamical systems analyses were applied to examination of postural sway, group differences became apparent in the medio-lateral but not the anterior-posterior direction. This finding of less complex dynamics in the BD group compared to controls is consistent with the loss of complexity hypothesis in disease and disorder [38], perhaps indicating weakened links between the sensorimotor systems, i.e., impaired integration of visual, vestibular, and proprioceptive systems, that form the critical feedback loops essential to the control of postural sway. Both groups demonstrated the expected pattern of results in response to manipulations of proprioceptive input. Specifically, DFA α-values increased when proprioceptive input was reduced (i.e., in the closed-base condition). Knowing that proprioceptive inputs contribute to short time-scale postural adjustments [43], this decrease in complexity, indicated by the increase in DFA α-values, most likely represents increased predominance of slow time-scale changes in posture that occur when proprioceptive information is reduced.

While the bipolar disorder and control groups had similar responses to manipulations of proprioceptive input (through changes in stance) overall, differences in sway dynamics between groups were particularly apparent when visual input was removed. Specifically, DFA α-values decreased for controls in the eyes-closed condition, but remained relatively unchanged in the bipolar disorder group. The DFA values for the BD participants remained high, indicating that their sway dynamics were dominated by slow time-scales of change. The pattern of results observed in the control group, in contrast, indicated that sway dynamics became more complex when visual input was removed, consistent with previous studies indicating that visual information contributes to low frequency, longer time-scale postural adjustments [41], [42], [43]. Therefore, removal of visual input would increase the relative contribution of short time-scales, resulting in more complex sway dynamics (i.e., reduced DFA α-values). This allows the short time-scale proprioceptive inputs to compensate for the absence of visual information by becoming the predominant means of generating postural corrections [43].

The fact that the bipolar disorder group maintained high DFA α-values (indicative of reduced complexity) even when visual input was removed suggests that the BD participants were less able to make corrections to their sway pattern when no visual information was available. Reduced short time-scale corrections contributes to decreased complexity in postural sway in BD, a finding that is consistent with the postulation that aging and disease are associated with a loss of complexity due to the loss of short time-scale components in physiologic systems [38]. One possible explanation for this result is that individuals with BD have a compressed range of time-scales available with which to make postural corrections, preventing them from making the shorter time-scale corrections that the controls were able to implement. Another possible explanation is that the BD participants have a reduced ability to integrate and utilize proprioceptive information for motor control. Interestingly, BD participants appear to be able to increase the contribution of slow time-scale postural corrections similar to controls, as they exhibited an increase in DFA α-values from the open-base to the closed-base. Overall, these findings converge to suggest that BD participants are restricted in their ability to adapt to task demands only if the task requires greater fast timescale postural corrections.

A consistent finding across the sway area and DFA analyses was that a large decline in postural control occurred in the eyes-closed condition in BD irrespective of stance. In particular, increased sway area and decreased ability to implement postural adjustments in the medio-lateral direction in the BD group were apparent compared to controls when visual input was removed. The convergence of our results across both magnitude and dynamic analysis is important, especially because dynamic analyses (such as DFA) are often considered a more sensitive assay of postural sway and are able to reflect different properties of postural control that sway area alone cannot [37], [53]. Furthermore, DFA α-values were less variable between subjects than the sway area (see Figure 3), which increases confidence in results obtained using both approaches.

It may be of significance that diagnostic group differences in postural dynamics were found only in the medio-lateral direction. In general, postural sway in the anterior-posterior direction is primarily generated at the ankle, while postural control in the medio-lateral direction is the product of hip movements [54], owed primarily to the anatomical properties of these joints. One interpretation of the present results is that they could be indicative of abnormal motor development in BD, given that motor development of the postural system often follows a distal-to-proximal direction (foot-to-hip). Developmental insults can alter the sequence of motor development [55]. Subtle developmental alterations are one possible explanation for the current results; in this context the BD participants may not have fully developed the control of posture using their hips. While speculative, this postulation is consistent with studies supporting a role of neurodevelopmental factors contributing to bipolar disorder [56], [57], [58], [59], [60] (but see [61]).

The results of this current study are consistent with previous observations of comorbidities between motor dysfunction and mood disorders. For example, in Parkinson's Disease, depression is a common feature of the illness [62], [63] and appears to increase in severity as PD progresses [64]. These findings suggest that mood dysregulation may be a core feature of the disease process in Parkinson's [3]. In Huntington's Disease, both depression [65] and mania [66] are commonly reported. Moreover, pathophysiological alterations in the circuitry implicated in depression have also been observed in Huntington's Disease, i.e., decreased glucose metabolism in orbitofrontal cortex and posterior parietal regions [67]. In addition, although the basal ganglia circuitry is primarily affected, there is also evidence of cerebellar abnormalities in both Parkinson's [68], [69] and Huntington's [70], [71], [72]. These comorbidities between motor and mood disorders suggest dysfunctions in similar neural circuits may underlie both types of pathology, although further research is needed to gain a greater understanding of the differences in symptom presentation across these disorders.

One complication in fully understanding the current results is the relative heterogeneity in the BD participants, as evidenced by the large between-subjects standard deviations in sway area. The heterogeneity in the BD group could have arisen as a result of differences in medication regimens. Medication confounds are difficult to completely eliminate or adequately control for statistically. Gaining access to medication naïve patients is also not a completely satisfactory answer because such patients are often symptomatic, introducing a confound of acute mood state. Therefore, testing medicated, euthymic patients represents one approach to investigating the underlying mechanisms of bipolar disorder. Testing never-medicated first-episode (often symptomatic) patients is a complementary strategy. Each approach presents a different type confound (medication vs. clinical mood state status), but nevertheless provides a part of the overall picture of the pathophysiology of BD.

The approach we have chosen for this study, i.e., studying euthymic, medicated patients, clearly presents difficulties in the interpretation of the present results because it is difficult to determine what proportion of the effect size arises from underlying mechanisms associated with bipolar disorder and what effects were due to medications. Although the small sample size makes disentangling medication effects difficult, an additional, possibly insurmountable obstacle is the number of different psychotropic medications each participant was on, often with different pharmacological mechanisms, and their interacting and sometimes opposing effects on postural control. For example, neuroleptics have been shown to negatively affect sway dynamics [73] while SSRIs have been shown to reduce the amount of sway in animal models [74], an effect that would be viewed as enhanced postural control.

In addition, postural sway may have been altered in patients taking lithium. There is evidence that lithium improves motor coordination and balance on the rotarod test in a transgenic mouse model of Huntington's disease [75]. Lithium is also believed to have neuroprotective effects in bipolar disorder [76] and such effects have been observed in animal models [77], [78]. Notably, lithium prevented apoptosis in the striatum in a rat model of HD [79] and cerebellar granule cell death [80], suggesting a mechanism by which it could improve motor function over the long-term. In the context of the present study, 4 of 16 BD patients were on lithium, which could have had a normalizing effect on the postural sway performance of these patients. However, we still observed significant between groups differences in spite of lithium treatment in 25% of our sample. Additional information about the existence of postural control deficits could be obtained by studying postural sway in a medication-naïve, or at least a currently unmedicated sample of bipolar disorder individuals. This would be a necessary step in order to obtain a more definitive answer to the question of whether postural control abnormalities exist in this population in the absence of any medications. However, as previously discussed, this approach comes with its own set of difficulties, i.e. the possible confound of acute clinical symptoms that have accompanying alterations in motor behavior.

In this current experiment, we do not have sufficient statistical power to clearly delineate how individual medications and course of illness variables such as the number of previous mood episodes could have affected postural control. This process is complicated further by the potentially broad range of effects that different combinations of medications prescribed to participants could have on motor function. Further longitudinal research with a better controlled, much larger sample is necessary in order to elucidate the different effects of various medications and their combined effects on postural sway in BD. In addition, more comprehensive information regarding the relationship between illness history and postural control would be of interest. Overall, however, comparison of participants based on categories of medication use does provide some evidence that the observed deficits in motor function cannot be explained as being the effects of the psychotropic drugs alone. It is possible that some interaction between the disorder and medications negatively impacts postural control in BD.

Several limitations to the present study suggest caution should be exercised in interpreting our results. Beyond medication use, several additional sources of sample variance in the BD group may have influenced the group differences that were observed. A number of BD participants in this study had a history of alcohol abuse or dependence, which could contribute to the observed differences between groups. However, the observed pattern of results was unchanged when participants with previous alcohol dependence were excluded. An additional source of variance is inter-individual differences in illness history. Such course of illness variables could be particularly relevant given that the number of previous acute mood episodes in bipolar disorder has been associated with the degree of cerebellar atrophy [19], especially in the posterior cerebellar vermis [13], [18], and with basal ganglia volume, especially in the putamen [60], [81].

Overall, the evidence presented here is consistent with earlier findings of motor abnormalities in BD [3], [5], [6], [7] and is consistent with the proposed deficits in the cerebello-striatal-prefrontal circuit [1], [2]. Although the literature in this area is limited, a picture is emerging in which mood and motor dysfunction are comorbid pathophysiological features with closely overlapping core components. Further research into the nature of motor abnormalities in BD is warranted, ideally with never medicated or currently unmedicated participants. Structural and functional neuroimaging studies conducted in conjunction with assessments of mood state and motor performance would be particularly informative as to the existence and characteristics of motor dysfunction in BD.
